# Supplementary material for: Gametes deficient for Pot1 telomere binding proteins alter levels of telomeric foci for multiple generations
Source: Commun Biol. 2021 Feb 4;4:158. doi: 10.1038/s42003-020-01624-7 (PMC7862594; doi:10.1038/s42003-020-01624-7)

Supplementary Figure 1

a-b) POT-1::mCherry foci in wild type (a) and pot-2 mutant (b) backgrounds. Oocyte position is indicated with yellow text. **c)** POT-1::m-Cherry in the distal germline of L1-L4 larvae and young adult worms. **d)** Quantification of POT-1::mCherry in distal germ cells of L1-L4 larvae and young adults. * $p < 0.05$, *** $p < 10-5$. **e)** Diagram of crosses using outcrossed pot-2 mutants with wild-type telomere lengths. **f)** pot-2 mutants possessing normal telomere lengths, corresponding to F4 embryos shown in panel e, have elevated levels of Pot1 foci in early embryos. Scale bars are 10 μm. Error bars are 95% confidence intervals.

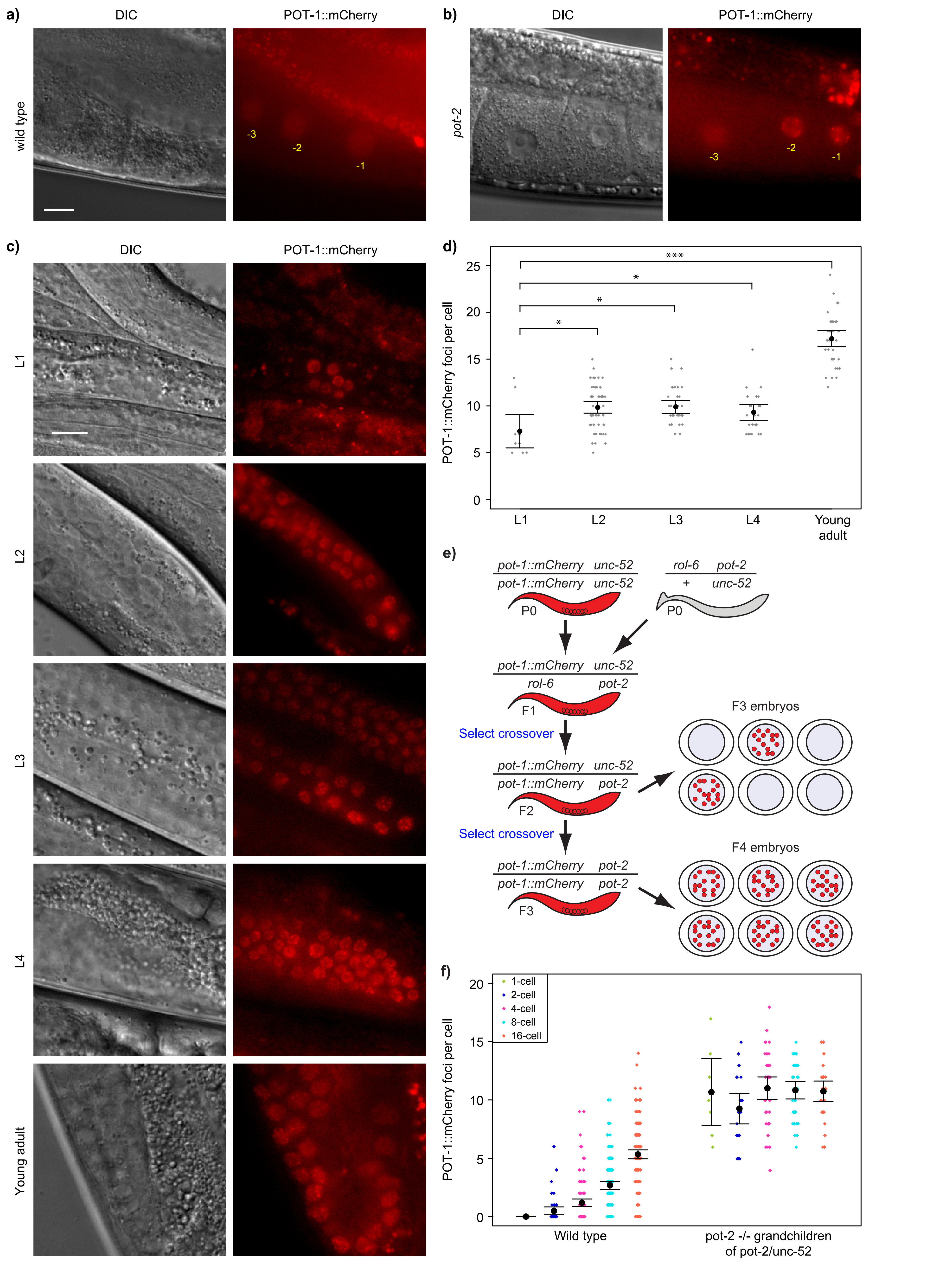

Supplementary Figure 2

a) A Southern blot of genomic DNA prepared from the indicated genotypes. **b)** DIC and fluorescent images of pot-1::mCherry mNeon-Green::pot-2 worms. Representative images from the germline mitotic zone, pachytene zone, oocytes, sperm, and embryos of early and late stages. **c)** Diagram of the experimental setup used to quantify Pot1 foci in the wild-type descendants of pot-2 mutants. **d)** Pot1 foci counts in the F2 descendants different pot-1 and pot-2 mutants. These independent mutations confirm results shown in panels 2a and 2c. **e)** mNeonGreen::POT-2 localization in the embryos and germline of pot-1 mutant homozygotes. **f)** F2 embryos obtained by crossing pot-1::mCherry mNeonGreen::pot-2 males to trt-1 mutant hermaphrodites that maintain their telomeres via ALT. **g)** POT-1::mCherry foci in embryos of homozygous trt-1 mutants propagated by ALT. Scale bars are 10 μm. Error bars are 95% confidence intervals.

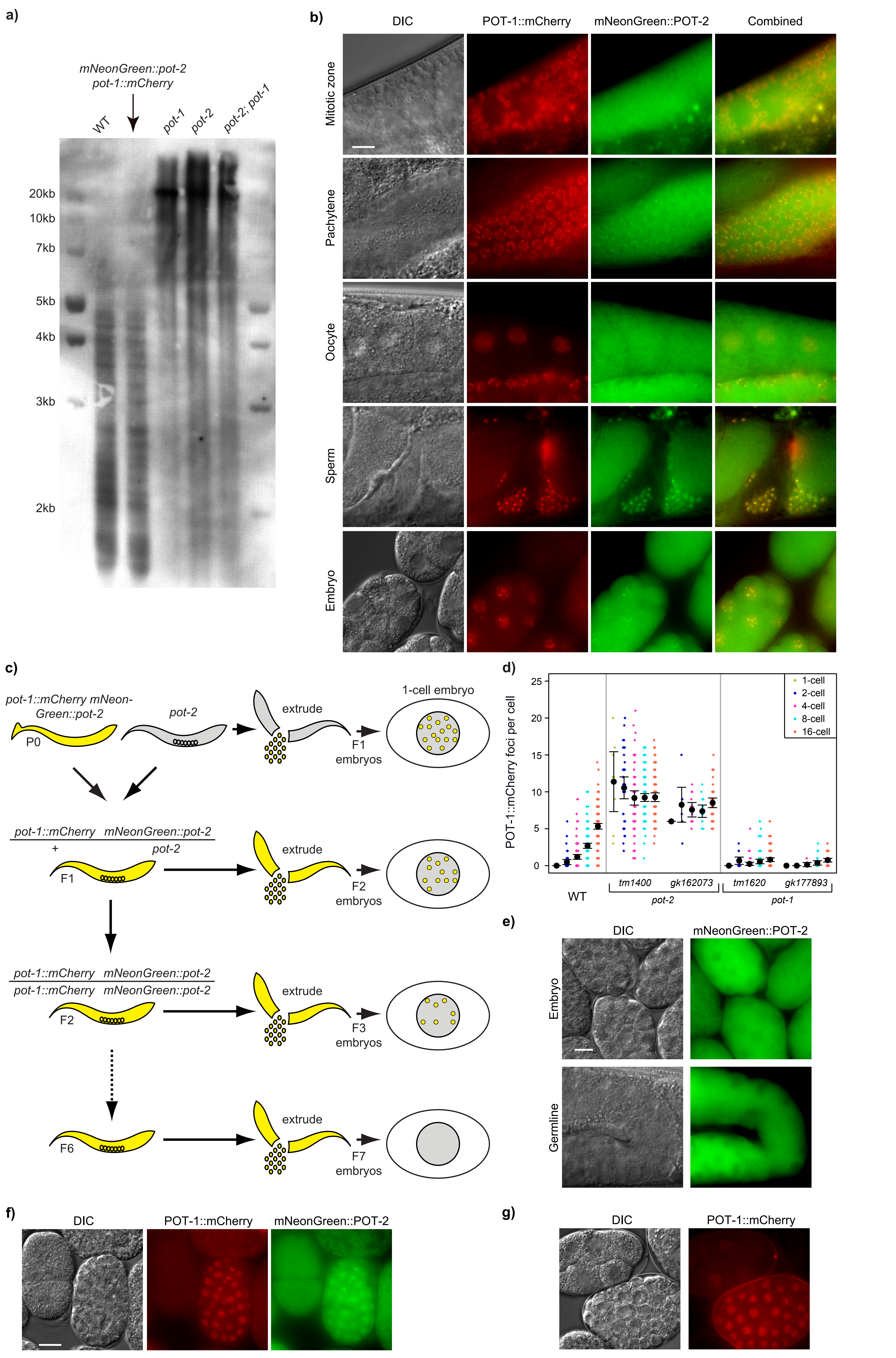

Supplementary Figure 3

a) Quantification of POT-1::mCherry foci in F2 embryos derived from crosses of pot-1::mCherry males with hermaphrodites mutant for the indicated alleles. These are independent alleles of mutations shown in Fig. 3b. **b)** Quantification of POT-1::mCherry foci in homozy-gous mutant backgrounds for the indicated genes. **c)** Southern blot using genomic DNA from met-2, set-25, and set-32 mutants. Error bars are 95% confidence intervals.

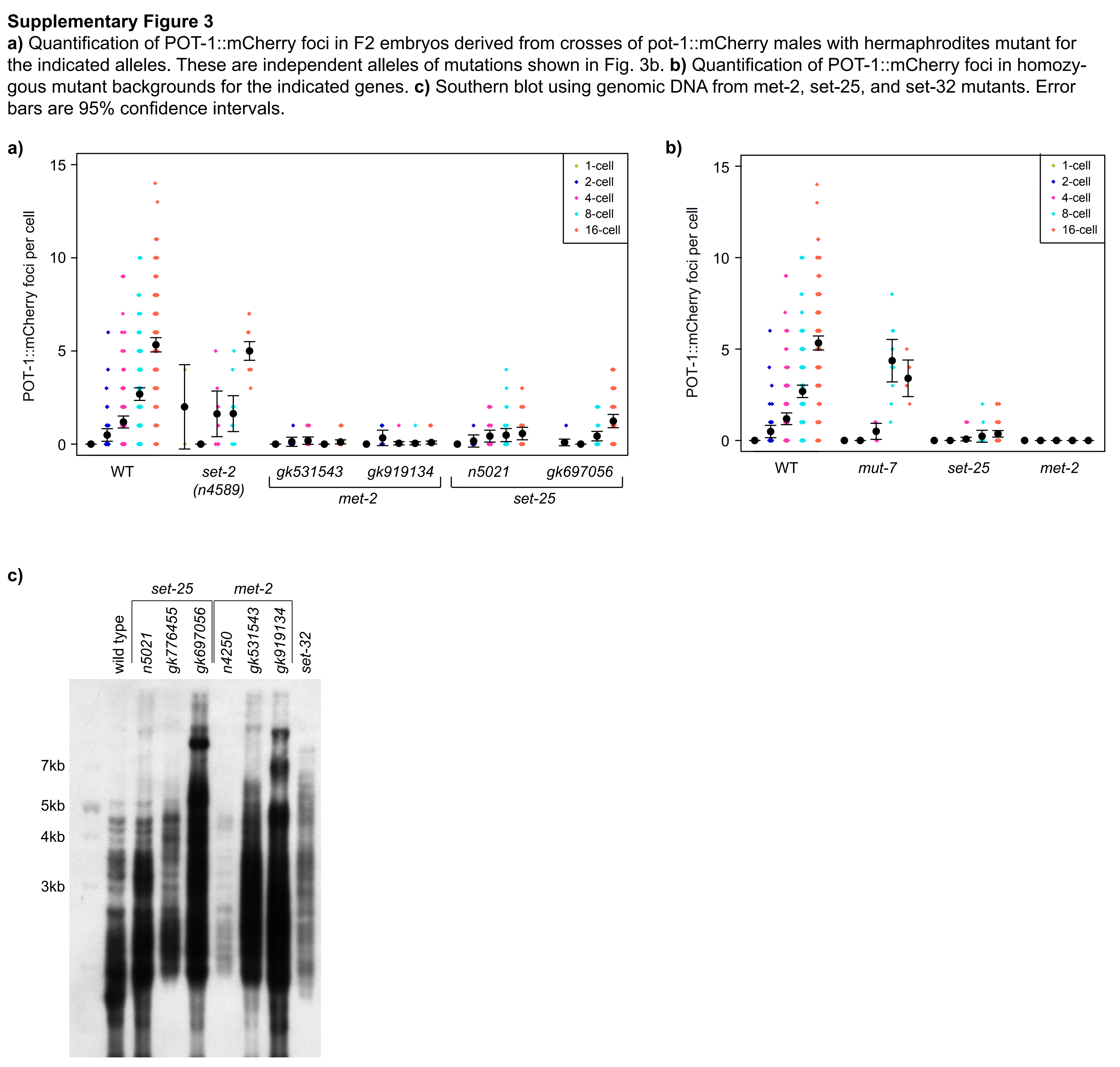

Supplement: Supplementary file 2 — Supplementary information [file 42003_2020_1624_MOESM2_ESM.pdf]
