# Supplementary material for: Gametes deficient for Pot1 telomere binding proteins alter levels of telomeric foci for multiple generations
Source: Commun Biol. 2021 Feb 4;4:158. doi: 10.1038/s42003-020-01624-7 (PMC7862594; doi:10.1038/s42003-020-01624-7)
Supplement: Supplementary file 3 — Description of Additional Supplementary Files [file 42003_2020_1624_MOESM3_ESM.pdf]

Description of Additional Supplementary Files

File name: Supplementary Data 1
Description: Excel file of sample sizes for each Pot1 foci quantification panel.
